# The most common founder pathogenic variant c.868G > A (p.Val290Met) in the *NPHS2* gene in a representative adult Czech cohort with focal segmental glomerulosclerosis is associated with a milder disease and its underdiagnosis in childhood

**DOI:** 10.3389/fmed.2023.1320054

**Published:** 2023-12-19

**Authors:** Dana Thomasová, Michaela Zelinová, Malgorzata Libik, Jan Geryk, Pavel Votýpka, Silvie Rajnochová Bloudíčková, Karel Krejčí, Jana Reiterová, Eva Jančová, Jana Machová, Martina Kollárová, Ivan Rychík, Martin Havrda, Miroslava Horáčková, Martina Putzová, Roman Šafránek, Marek Kollár, Milan Macek

**Affiliations:** ^1^Institute of Biology and Medical Genetics, University Hospital Motol and 2nd Faculty of Medicine, Charles University Prague, Prague, Czechia; ^2^Department of Nephrology, Institute for Clinical and Experimental Medicine, Prague, Czechia; ^3^3rd Department of Internal Medicine-Nephrology, Rheumatology and Endocrinology, University Hospital and Faculty of Medicine Palacký University Olomouc, Olomouc, Czechia; ^4^Department of Nephrology, General University Hospital in Prague, First Faculty of Medicine, Charles University, Prague, Czechia; ^5^Department of Internal Medicine I, Faculty of Medicine in Pilsen, Charles University and Teaching Hospital, Pilsen, Czechia; ^6^Biomedical Centre, Faculty of Medicine in Pilsen, Charles University, Pilsen, Czechia; ^7^Department of Internal Medicine, University Hospital Vinohrady and Third Faculty of Medicine, Charles University, Prague, Czechia; ^8^Department of Internal Medicine, University Hospital Motol and 2nd Faculty of Medicine, Charles University, Prague, Czechia; ^9^Biopticka Laboratory, Pilsen, Czechia; ^10^Faculty of Medicine in Plzeň - Charles University, Pilsen, Czechia; ^11^Department of Nephrology, University Hospital Hradec Králové, Faculty of Medicine in Hradec Králové, Charles University, Hradec Králové, Czechia; ^12^Department of Pathology, Institute for Clinical and Experimental Medicine, Prague, Czechia

**Keywords:** end-stage renal disease, exome sequencing, focal segmental glomerulosclerosis, nephrotic syndrome, *NPHS2*, proteinuria

## Abstract

**Background:**

Genetic focal segmental glomerulosclerosis (FSGS) is caused by pathogenic variants in a broad spectrum of genes that have a variable representation based on subjects' ethnicity and/or age. The most frequently mutated autosomal recessive gene in FSGS is *NPHS2*. In this study, we analyzed the spectrum of *NPHS2* variants and their associated phenotype in Czech adult FSGS patients.

**Methods:**

A representative cohort of 234 adult patients with FSGS, derived from 225 families originating from all regions of Czechia, was analyzed by massively parallel sequencing. In this study, we focused on the comprehensive analysis of the *NPHS2* gene. The histological classification of FSGS followed the Columbia classification.

**Results:**

We detected seven (3%) cases bearing homozygous or compound heterozygous pathogenic *NPHS2* variants. A single pathogenic variant c.868G > A (p.Val290Met) was found in the majority of *NPHS2*-positive cases (86%; 6 out of 7) in histologically confirmed instances of FSGS. Its allele frequency among unrelated *NPHS2*-associated FSGS patients was 50% (6/12), and Haplotype analysis predicted its origin to be a result of a founder effect. There is an identical V290M-related haplotype on all V290M alleles spanning a 0,7 Mb region flanking *NPHS2* in Central European FSGS populations. The phenotype of the p.Val290Met *NPHS2*-associated FSGS demonstrated a later onset and a much milder course of the disease compared to other *NPHS2* pathogenic variants associated with FSGS. The mean age of the FSGS diagnosis based on kidney biopsy evaluation was 31.2 ± 7.46 years. In 50% of all cases, the initial disease manifestation of proteinuria occurred only in adulthood, with 83% of these cases not presenting with edemas. One-third (33%) of the studied subjects progressed to ESRD (2 out of 6) at the mean age of 35.0 ± 2.82 years.

**Conclusions:**

We identified the most prevalent pathogenic variant, p.Val290Met, in the *NPHS2* gene among Czech adult FSGS patients, which has arisen due to a founder effect in Central Europe. The documented milder course of the disease associated with this variant leads to the underdiagnosis in childhood. We established the histopathological features of the *NPHS2*-associated adult FSGS cases based on the Columbia classification. This might improve patient stratification and optimize their treatment.

## Introduction

Focal segmental glomerulosclerosis (FSGS; MIM: 603278; ORPHA: 656 with synonyms Familial Idiopathic Steroid-Resistant Nephrotic Syndrome/SRNS/; Genetic SRNS; Hereditary SRNS) represents a heterogeneous spectrum of clinical conditions that have distinct histopathological kidney findings in renal biopsy (1). It is characterized by segments of sclerosis in a section of the glomerular tuft but only in some glomeruli (2). Histopathologic “Columbia classification” distinguishes five morphological FSGS subgroups (3–5).

The hallmark clinical symptom of FSGS is proteinuria, which may vary from mild to nephrotic-range proteinuria. FSGS is one of the most frequent causes of nephrotic syndrome (NS), characterized by hypoalbuminemia, hyperlipidemia, and edema in adults. NS commonly results in end-stage renal disease (ESRD) that requires kidney replacement therapy, i.e., hemodialysis or kidney transplantation (6).

FSGS is the result of various pathogenic processes that damage podocytes. FSGS can be classified into secondary, primary, and genetic forms (1). Secondary FSGS occurs due to known stress, toxic, immunologic, or viral factors that damage podocytes, for example, hypertension, obesity, or several nephrotoxic medications. Primary FSGS is caused by an unidentified “circulating factor,” which negatively affects podocytes and thus increases the permeability of the glomerular filtration barrier (7). Genetic FSGS exhibits a high underlying genetic heterogeneity and is considered a rare monogenic disorder. This classification is attributed to pathogenic variants (henceforward mutations in legacy nomenclature) found in a broad spectrum of genes encoding proteins implicated in upholding structural and functional stability, as well as homeostasis, of podocytes and the glomerular basement membrane (8). Over the last several decades, variants in more than 50 genes were implicated as causative for FSGS (9, 10). These variants are transmitted both in the autosomal recessive and autosomal dominant manners.

Since the treatment of different forms of FSGS requires customization, i.e., therapeutic approaches differ in genetic vs. non-genetic FSGS, it is important to identify cases with genetic forms of FSGS that usually do not adequately respond to corticosteroid-based therapy and subsequently do not reach complete remission (10, 11). Therefore, monogenic forms of FSGS can manifest as steroid-resistant nephrotic syndrome (SRNS; ICD-10 code N04; henceforward we shall use only the FSGS abbreviation for nomenclature clarity).

The mutations in the *NPHS2* gene (MIM: 604766), having an autosomal recessive inheritance pattern, are the most frequent cause of early childhood-onset FSGS. In the European and North American pediatric series, the prevalence of FSGS due to the *NPHS2* mutations ranges from 4 to 30% in familial cases and from 0 to 11% in sporadic cases (12, 13). The predominant *NPHS2* pathogenic variant in European pediatric cohorts is the European founder mutation p.Arg138Gln, with the early onset of NS symptoms (median age of 17 months) and rapid progression to ESRD within the first decade of life (14). In Western Europe, the most frequent genotype associated with adult FSGS consists of compound heterozygous mutations in the *NPHS2* gene most often comprising the variant p.Arg229Glu (R229Q in legacy nomenclature) together with another pathogenic variant in exon 7 or 8 (15) in the *NPHS2* gene *in trans*. This genotype is found in almost 95% of adult FSGS patients in the aforementioned region (12, 16, 17). Podocin (Swiss-Prot: Q9NP85), the protein product of the *NPHS2* gene, is a crucial part of the slit-diaphragm (SD) of the glomerular filtration barrier. Podocin binds nephrin and other SD proteins to the cytoskeleton (18).

We conducted a comprehensive molecular genomic study of a representative Czech cohort with clinically diagnosed forms of FSGS, characterized by typical histopathologic findings obtained from kidney biopsies. We conducted a whole exome analysis, analyzed variants in previously reported disease-causing genes (data not shown), and focused on the spectrum of variants within the *NPHS2* gene. Hereby, we present the identification of a founder mutation c.868G > A (p.Val290Met) in the *NPHS2* gene, specific for the representative Czech population of adult FSGS patients, accounting for 83% of all patients with *NPHS2* mutations. We describe genotype-phenotype correlations, including histopathological findings in our cases. To date, this is one of the largest and most comprehensive genetic analyses of a Central European FSGS population.

## Methods

### Patients

We analyzed a representative cohort of 234 Czech adults with FSGS drawn from 225 families (aged 18 to 85 years), with a mean age of 47.0 ± 18.3 years. Patients were recruited by adult nephrologists from collaborating university hospitals which routinely performed diagnostic kidney biopsies in suspicious cases of proteinuric disease. The participating Czech centers comprise seven university hospitals (Motol, General University Hospital, Kralovské Vinohrady, Institute of Clinical and Experimental Medicine, Pilsen, Hradec Kralové and Olomouc) covering tertiary centers from the entire country. The multidisciplinary teams provided clinical data, signed informed consent forms for participation in the study, and blood samples from index cases compiled in the National Kidney Biopsy Registry (https://www.nefrol.cz/odbornici/registry), and nephrologists followed the patients in their outpatient clinics. FSGS was defined by histopathological (3), clinical, and laboratory findings, according to the aforementioned diagnostic criteria. Patients with secondary FSGS were excluded from the study. The inclusion criteria were as follows: 1. adult FSGS patients with a positive family history, where the diagnosis was confirmed by kidney biopsy in at least one family member; and 2. primary FSGS resistant to steroid therapy, i.e., FSGS/SRNS following medication with 1 mg/kg of prednisone in a 6-month treatment scheme. The selected subjects were referred to the Department of Biology and Medical Genetics at Motol University Hospital Prague (www.fnmotol.cz/ublg), which hosts an ISO15189:2013 accredited genomics facility where they underwent genetic counseling before signing a standardized informed consent for (whole) exome sequencing (ES). All cases were offered clinical genetic examination within a three-generation genealogy in the case of identification of a pathogenic variant in the index case. Relevant family members were provided the option for examination to determine the presence of the respective variant, with preconception care offered in selected cases.

The study was approved by the Internal Ethics Board of the Motol University Hospital within the framework of the Czech Medical Research Foundation project. It was carried out as per the provisions of Art. 28-29 of Act 373/2011 Coll. and adhered to the principles of the World Medical Association's “Declaration of Helsinki.” This research study was designed in order not to include/report secondary findings from ES data (as per the latest American College of Medical Genetics guidelines; ACMG.net; https://www.ncbi.nlm.nih.gov/clinvar/docs/acmg/) in all study subjects due to legal restrictions outlined in the aforementioned Czech legislation.

### Massively parallel sequencing

Template genomic DNA was obtained from peripheral blood samples (stored with K3EDTA anticoagulant) using a MagCore™ HF16 Plus automated nucleic acid extractor (RBC Bioscience; Republic of China). DNA concentration was measured using the NanoDrop™ 2000 spectrophotometer (Thermo Scientific; USA) and Qubit™ 2.0 devices (Invitrogen, USA), according to the manufacturer's instructions. The library preparation for massively parallel-based ES was performed in all cases using three assays (Roche MedExome™ – Roche, USA; Twist™ Core Exome – TWIST Bioscience, USA; SOPHiA DDM™ Whole Exome Solution – SOPHiA GENETICS, Switzerland). The library for WGS sequencing was prepared using the TruSeq DNA PCR-free assay (Illumina; USA). All libraries were sequenced on a NovaSeq X Plus and/or NovaSeq6000 platforms (Illumina, USA) for 150 cycles in a paired-end mode in collaboration with the Institute of Applied Biotechnologies, Prague, Czechia (www.iabio.eu). All ACMG.net Class 4 and 5 variants (i.e., likely pathogenic and pathogenic, respectively) were validated through targeted Sanger DNA sequencing, and cascade family screening was performed where applicable to confirm segregation and determine the linkage phase of the detected mutations. In an extended bioinformatical analysis, we systematically searched for pathogenic/likely pathogenic variants in 165 genes associated with kidney disease, including those currently listed in Online Inheritance in Man™ (OMIM.org) related to NS/FSGS (Supplementary Table 1). This also encompassed genes associated with proteinuric renal diseases (see Results).

### Data processing and variant annotation

The sequencing (fastq) data were mapped to the human hg38 genome using the “processing-for-variant-discovery-gatk4” pipeline and the basic quality analysis was performed. The absolute majority of bases had quality scores above Q35, with minimum coverage above 10. The single nucleotide polymorphism (SNP) and indel variants were detected by the “haplotypecaller-gvcf-gatk4” and the joint variant calling was performed using the “joint-discovery-gatk4.wdl” pipelines. All above-mentioned pipelines are freely available at the Broad Institute git repository: https://github.com/gatk-workflows. The resulting joint vcf was annotated by an in-house pipeline based on the Annovar annotation program (https://annovar.openbioinformatics.org/en/latest/). The above-defined procedure applies to both ES and whole-genome sequencing (WGS) data in the selected ES-negative cases (NovaSeq Reagent Kit ver. 1.5; Illumina, USA, Illumina.com). In addition to the in-house annotation pipeline, ES data were also annotated by the BaseSpace Variant Interpreter™ (Illumina, USA, Illumina.com) and SOPHiA DDM™ Platform (SOPHiA GENETICS, Switzerland, Sophiagenetics.com). The structural variants were identified by the software Manta (https://github.com/Illumina/manta) in the case of WGS data.

### Variant filtering and evaluation

To assure maximum quality of bioinformatic analyses and account for potential biases in each available pipeline, mainly in terms of tertiary analysis, prioritization of detected variants from ES was performed by compiling the outcomes of four independent bioinformatics pipelines, comprising (a) the in-house developed filtering pipeline (specifications are available upon request), (b) the BaseSpace Variant Interpreter (Illumina, USA; https://variantinterpreter.informatics.illumina.com/home), (c) the SOPHiA DDM™ Platform (https://www.sophiagenetics.com/technology/sophia-ddm-for-genomics/; Switzerland), and (d) the Varsome Clinical software (SAPHETOR, Switzerland; https://landing.varsome.com/varsome-clinical), to select consistently identified likely pathogenic/pathogenic variants that were visualized and assessed using the Integrative Genomics Viewer™ (IGV; Broad Institute; USA) and Alamut™ Visual Plus (SOPHiA GENETICS, Switzerland). The prioritization was based on their prevalence in the general “European non-Finnish population” (as documented in gnomAD, https://gnomad.broadinstitute.org/, and dbSNP, https://www.ncbi.nlm.nih.gov/snp/), their presence in clinical databases (e.g., ClinVar, HGMD), interspecies residue conservation and coherence, familial cosegregation with the selected phenotype, and *in silico* predictions using bioinformatics tools integrated within the Varsome Clinical software (DANN, DEOGEN2, EIGEN, FATHMM-MKL, M-CAP, MVP, MutationAssessor, MutationTaster, PrimateAI, REVEL, PolyPhen, and SIFT). The in-house filtration pipeline proceeded as follows: In the first step, we retained only variants belonging to any of the 165 genes associated with nephropathy, filtering for those with a frequency of 0.01 or lower in the Genome Aggregation (gnomad) database. Subsequently, in the second step, only non-synonymous or splicing variants or variants with any annotation from RefSeq, Ensembl, Encode, and Oregano regulatory databases remained in the final call set after the filtration procedure. In individual patients, the variants within the final call set were evaluated one by one for pathogenicity and assessed for their correlation with the patient's phenotype. The vcf files from Manta were filtered by selecting all structural variants overlapping the coding structure of any of the 165 genes associated with kidney disease (Supplementary Table 1). Subsequently, they were individually evaluated. All patient sequencing data were primarily screened for *NPHS2* Class 4 and 5 variants (19), variants of unknown significance (VUS), and the non-neutral polymorphism c.686 G > A (p.Arg229Gln). For WGS data, intronic variants with potential regulatory impact on the *NPHS2* gene were also assessed. We also utilized two Czech population genomic cohorts: (a) “ENIGMA,” consisting of 677 unrelated healthy individuals (https://czechgenome.iabio.eu/), and (b) the “Analysis of Czech Genomes for Theranostics” cohort (ACGT; https://database.acgt.cz), comprising 1,016 random healthy subjects. Both cohorts underwent WGS for the assessment of population-specific variant frequencies. However, we could not include these cohorts in the founder mutation identification pipeline (see below) due to the proprietary restrictions of these datasets containing only variant frequencies in apparently healthy Czech subjects.

### Predictions of founder mutations associated with FSGS

We defined potential founder mutations associated with FSGS as a variant that is significantly overrepresented within the FSGS cohort vs. the control population. We used 649 probands as a control population composed of anonymous healthy cases (430; random controls) collected previously for other studies and 219 patients with confirmed genetic diseases affecting various organ systems (e.g., from cardiogenetic- or deafness-related studies) without any kidney disease.

Any type of genetic relatedness was eliminated from both FSGS and control groups before the prediction of founder mutation. Only the index cases were retained in the FSGS group to eliminate the potential identification of family-shared FSGS-unrelated variants as false positive founder mutations. In addition, variants with allelic fraction <0.3 were removed from both groups to reduce the number of false positive founder mutations caused by artificial variants. We used two statistical models for predicting the founder mutation. The first model defined the founder mutation as a variant with an odds ratio (OR) >0.5 with a confidence interval around the estimate of OR that did not include 1. This definition is equivalent to the PS4 rule within the ACMG classification system. The second model was based on the hypergeometric distribution. The variant frequency within the control population was used to estimate the expected number of mutation carriers in the general Czech population. The probability of having the same or higher number of mutation carriers within the random subset from the Czech population as in the FSGS cohort was then calculated using the hypergeometric model. Both statistical models were applied to all variants within the final call set and resulted in *p*-values corrected by the Bonferroni correction.

### Haplotype analysis

To determine the haplotype that carried the mutated allele, haplotype analysis was initially performed in two families with an affected member and carrying parent, and the results were compared with eight affected individuals carrying the commonly identified c.868G > A mutation in the FSGS (see further details in the Results section). We used 10 markers surrounding the *NPHS2* gene, namely, D1S416, D1S212, D1S2786, D1S460, and D1S2640 (located 5′) and D1S215, D1S2751, D1S2883, D1S2818, and D1S466 (located 3′) (Supplementary Table 2). The analysis was performed through PCR using fluorescent-labeled primers (FAM, PET, VIC, and NED; https://www.thermofisher.com/order/custom-oligo/fluorescent-labelled-primers) followed by capillary electrophoresis using the Applied Biosystems™ 3130xl Genetic Analyzer (Thermo Fisher Scientific; USA). The results were analyzed and compared using GeneMapper™ software version 6 (https://www.thermofisher.com/order/catalog/product/4475073). We identified a minimum shared area of approximately 0.7 Mb confined by the markers D1S2640, D1S215, and D1S2751 surrounding the *NPHS2* gene.

### Histopathology analyses of kidney biopsies

Renal biopsies were evaluated histopathologically by a standard protocol according to recent professional guidelines (20). Immunofluorescence, light microscopy, and electron microscopy analyses were performed on the bioptate. Immunofluorescence examined immunoglobulins (IgG, IgA, IgM), complement components (C3, C1q), light chains (kappa and lambda), serum amyloid A, and monoclonal antibodies recognizing the collagenous domain of alpha1 (MAB1), alpha3 (MAB3), and alpha5 (MAB5) chains of type IV collagen. The following pathology parameters, such as the type of focal segmental glomerulosclerosis, total/sclerotic number of glomeruli, interstitial fibrosis and inflammation, vascular changes, and fusion of foot process effacement, were assessed. The type of FSGS in biopsies was evaluated based on the current “Columbia Classification,” distinguishing five types of glomerular FSGS lesions, such as the collapsing variant, tip variant, cellular variant, perihilar variant, and FSGS not otherwise specified (4, 5).

## Results

### *NPHS2* gene variants

In the cohort of 234 adult patients from 225 families with confirmed FSGS through histopathological findings in kidney biopsy, we detected seven (3%) patients with homozygous or compound heterozygous pathogenic variants in the *NPHS2* gene. Notably, six out of these seven patients carried the c.868G > A (p.Val290Met) *NPHS2* pathogenic (Class 5, according to ACMG with supporting evidence from PM1, PM2, PP3, PS1) variant.

Two related patients (209/I and 209/II) carried this mutation in a homozygosity. In the two unrelated probands (144/I and 78/I), the c.868G > A variant was combined with the c.412 G > A *NPHS2* variant in *trans*. Another patient, 115/I, was found to have the c.868G > A variant along with a novel c.253dupG variant. Furthermore, we identified an additional six patients who carried the c.868G > A variant in *NPHS2* in the heterozygous constitution but without *trans*-association with another *NPHS2* mutation in ES data. Subsequently, we subjected these six patients to WGS and identified a *trans*-associated deletion of exon 2 in patient 109/I. The exon 2 deletion of the *NPHS2* gene was the only finding revealed by WGS that was not detectable by ES in these cases. After selecting only snp/indel variants within the *NPHS2* gene, including introns and regulatory regions from the above-defined call set in the case of WGS data (see Methods), only two variants remained - one being the c.868G>A and the other an intergenic variant annotated as a promoter-flanking variant. We evaluated this variant as irrelevant because the regulatory signal was very weak. In an extended bioinformatic analysis of 165 proteinuria-associated kidney disease genes (see Methods), we did not detect any pathogenic/likely pathogenic variants having the autosomal-dominant inheritance pattern. With regards to Class 4 and 5 variants in respective autosomal recessive genes, only in the case of patient 75/I did we detect a heterozygous pathogenic variant NM_000151.3(G6PC1):c.247C > T p.(Arg83Cys); Class 5 in the gene *G6PC* (MIM: 613742) associated with autosomal recessive glycogen storage disease. In all other analyzed FSGS cases, VUS (Class 3) were detected within all the remaining NS-related genes tested (gene-specific data are available upon request).

The allelic frequency of the c.868G > A variant within the studied FSGS cohort (including only index cases) is 2.66% (12/450 alleles), while within the control group, it is present at 0.15% (2/1,298 alleles). The frequency of this variant within the ENIGMA population genomic cohort is 0.52% (7/1,354 alleles) and within the ACGT cohort, it is 0.39% (8/2,032 alleles); however, as indicated in the Methods section, we could not use these data for founder mutation identification.

The c.868G > A variant is the most significantly predicted founder mutation based on the available control data and both applied statistical models. In the case of the OR model, we obtained values of OR = 5.17, CI = < 3.3; 69.76>, and a Bonferroni corrected *p*-value of 0.0035. In the case of the hypergeometric model, we obtained a Bonferroni corrected *p*-value of 0.00000257. Both values indicate that this variant is significantly overrepresented within the FSGS cohort in comparison with the random control population.

Interestingly, we identified only one FSGS patient with a different combination of *NPHS2* mutations who carried a variant c.902C > A associated with the non-neutral polymorphism c.686G > A. The polymorphism c.686 G > A was highly prevalent in the adult FSGS cohort; it was detected in 29 patients (13.0%) but was not associated with another pathogenic *NPHS2* variant. In the control cohort consisting of 649 persons, we found polymorphism c.686G > A in 55 individuals, i.e., at 8.49%. The allelic frequency of the c.686G > A variant within the FSGS cohort was 6.5% and within the control group, it was 4.3%. The frequency of the variant within the independent ENIGMA and ACGT cohorts was 3.7% (50/1354 alleles) and 5.17% (105/2,032 alleles), respectively. The differences in the enrichment of this polymorphism between the FSGS cohort and the aforementioned population control groups were not statistically significant.

### Haplotype analysis

To investigate further the possible founder effect of the c.868G > A mutation in the Czech population, we performed the haplotype analysis in the two families with affected index patients 209/I (c.868G > A homozygote) and 115/I (compound c.868G > A heterozygote), including their parents. To expand the analysis, we also genotyped the alleles of the six individuals with c.868G > A in heterozygosity. We covered 10 markers surrounding the *NPHS2* gene (see Methods). We observed an identical c.868G > A-related haplotype (at markers D1S2640, D1S215, and D1S2751 flanking *NPHS2*) in all 12 c.868G > A alleles in *cis*, spanning approximately a 0.7 Mb conserved region. The non-c.868G > A-related alleles were all different from each other (data not shown). The haplotype analysis result supports our hypothesis that the c.868G > A mutation originates from a common Central European ancestor.

### Clinical features of the patients bearing the *NPHS2* mutations

The clinical characteristics of the patients with *NPHS2* c.868G > A mutations are summarized in Table 1. The only patient who did not carry the c.868G > A mutation and whose FSGS genetic disease was determined by the combination of *NPHS2* pathogenic c.902C > A (p.Ala301Asp) variant with the polymorphism c.686G > A in *trans* presented with proteinuria from the age of 1 year. The patient did not present with NS at the onset of the disease. She had persistent proteinuria throughout childhood and young adulthood. At the age of 23, she developed edema and nephrotic proteinuria reaching 8 g/24 h. The first kidney biopsy at the age of 25 was inconclusive, while the re-biopsy performed at the age of 27 showed FSGS. The patient did not respond to steroid, cyclophosphamide, or tacrolimus treatments and only achieved transient partial remission with cyclosporine therapy. The patient progressed to ESRD at the age of 38 and underwent a kidney transplantation without the recurrence of the disease. The slow progression of the disease corresponds to the FSGS course previously described in association with the disease caused by the *NPHS2* pathogenic variant combined with p.Arg229Gln polymorphism (12).

**Table 1:** Genetic and clinical characteristics of the patients with the *NPHS2* c.868G > A variant.

**Family number**	**Genetic diagnosis established**	**Nucleotide change**	**Amino acid change**	**Zygosity**	**Gender**	**Age at first symptoms (PU)**	**μHU**	**Renal biopsy diagnosis/age**	**PU (g/day)/edema at onset**	**CKD/ESRD/Tx (age)**
144/I	Yes	• c.868G > A • c.413G > A	• p.Val290Met • p.Arg138Gln	Comp. heterozyg.	F	Childhood	No	FSGS/35 y	11/N	G1A2 (36 y)
78/I	Yes	• c.868G > A • c.413G > A	• p.Val290Met • p.Arg138Gln	Comp. heterozyg.	F	24 y	No	• MCD/24 y • FSGS/25 y	Unknown/N	ESRD/Tx (37 y)
115/I	Yes	• c.868G > A • c.253dupG	• p.Val290Met • p.Glu85Glyfs^*^18	Comp. heterozyg.	F	3 y	No	• MPGN/3 y • MCD/14 y • FSGS/40 y	1/N	G1A3 (46 y)
109/I	Yes	• c.868G > A • del. Exon 2	p.Val290Met p. ?	Comp. heterozyg.	F	41 y	Yes	FSGS/41y	3/Y	G4A3 (43 y)
209/I	Yes	c.868G > A	p.Val290Met	Homozyg.	M	Childhood	Yes	• MCD/11 y • FSGS/15 y	13/N	ESRD/Tx (33 y)
209/II	Yes	c.868G > A	p.Val290Met	Homozyg.	M	18 y	No	Not done	Unknown/N	G1A2 (51 y)
209/III	No	c.868G > A	p.Val290Met	Heterozyg.	M	68 y	Yes	Not done	3.5/Y	G1A3 (74 y)
123/I	No	c.868G > A	p.Val290Met	Heterozyg.	M	39 y	Yes	FSGS/39 y	5/Y	G1A1 (43 y)
68/I	No	c.868G > A	p.Val290Met	Heterozyg.	F	72 y	Yes	FSGS/72 y	17/Y	G1A1 (80 y)
104/I	No	c.868G > A	p.Val290Met	Heterozyg.	M	63 y	No	FSGS/63 y	4/Y	G1A1 (78 y)
75/I	No	c.868G > A	p.Val290Met	Heterozyg.	M	25 y	No	FSGS/25 y	9.45/Y	G3A2 (36 y)
142/I	No	c.868G > A	p.Val290Met	Heterozyg.	F	27 y	Yes	AS/FSGS 27 y	Unknown/N	ESRD (39 y)

Comp. heterozyg., compound heterozygote; Homozyg., homozygote; y, years; μHU, microscopic hematuria; FSGS, focal segmental glomerulosclerosis; MCD, minimal change disease; MPGH, membranoproliferative glomerulonephritis; AS, Alport syndrome; PU, proteinuria; Y, yes; N, no; CKD, chronic kidney disease stage according to UK Kidney Association (https://ukkidney.org/health-professionals/information-resources/uk-eckd-guide/ckd-stages); ESRD, end-stage renal disease; Tx, kidney transplant. p. ? - amino acid change unknown.

The group of six FSGS patients bearing the most prevalent c.868G > A mutation in homozygosity or compound heterozygosity showed high intrafamilial/interpersonal variability of clinical symptoms regardless of the type of the *NPHS2* mutation on the other allele. The mean age of the FSGS diagnosis based on kidney biopsy evaluation was 31.2 ± 7.46 years. Three patients (3/6) presented with proteinuria already in childhood, while in the other three cases, their proteinuria started in adulthood with a mean age of 27.7 years. Furthermore, five out of the six patients did not present with edemas, i.e., fully developed NS, at the onset of their disease. All patients were resistant to 6-month immunosuppressive therapy. Furthermore, two of the six patients progressed to ESRD at age 30 and 37 years, respectively.

To exemplify the clinical heterogeneity even within the same family, we describe here the clinical course of the disease in two brothers (patients 209/I and 209/II) carrying the homozygous *NPHS2* c.868G > A mutations. Patient 209/I was diagnosed at the age of 3 years with nephrotic proteinuria without edema at the regular preventive medical check-up. During childhood and young adulthood, he experienced repeated full NS-related attacks resistant to immunosuppressive therapy. He reached ESRD at the age of 30 years and underwent a kidney transplantation without recurrence of the disease. The other sibling, 209/II, was diagnosed with proteinuria at the age of 18 years, never developed NS, and had not progressed to ESRD. Currently, at the age of 51, he is being monitored by his general practitioner for mild proteinuria, with normal renal function.

In contrast to these findings, the patients carrying only one *NPHS2* c.868G > A variant in the heterozygosity without another mutation in *trans* had the onset of their disease much later at the mean age of 45.2 ± 14.8 years. The first symptom in five of six patients (83%) was the fully developed NS with edemas unlike in the group of FSGS patients with two pathogenic variants in the *NPHS2* gene. All patients carrying *NPHS2* c.868G > A variant on one allele responded to corticosteroid or corticosteroid + cyclosporine therapy with partial remission of proteinuria.

### Histopathology analyses of kidney biopsies in patients bearing the c.868G > A mutation

Renal biopsy was performed on 10 patients with the c.868G > A mutation: five of the patients had two pathogenic variants in the *NPHS2* gene and the remaining five patients carried the mutation in heterozygosity. Standard immunofluorescence yielded completely negative results; in some instances, it showed very weak positivity for IgM or/and C3 staining in sclerotisations. Morphology of focal segmental glomerulosclerosis was present in kidney biopsy samples of all patients, nevertheless, they differed in the type of sclerotisations. The detailed histopathological findings are summarized in Table 2.

**Table 2:** Histopathological characteristics of the kidney biopsies of the patients with the c.868G > A variant in the *NPHS2* gene.

**Case**	**Type of FSGS**	**Another disease**	**Glomeruli**			**Interstitium**		**Vascular pathology**		**Foot process effacement**
			**Total**	**Globally sclerotic**	**Focally sclerotic**	**Fibrosis %**	**Infl**.	**cv**	**ah**	
144/I	NOS	N	12	1	6	10	No			Yes, diffuse
78/I	PH, NOS	N	4	0	2	X, MF	No	X	0–1	Yes, diffuse
115/I	X	N	5	2	0	< 10	Yes			Yes, diffuse
109/I	TL, PH	N	10	5	3	>50	Yes	1	1–2	Yes, diffuse
209/I	PH	N	12	7	1	10	Yes	0	1	Yes, diffuse
123/I	TL	N	9	1	3	< 5	No	0–1	0–1	Yes, diffuse
68/I	PH	DM GP	14	2	3	10	No	1	1	Yes, diffuse
104/I	X, MF	N	3	1	2	X, MF	No	0–1	1–2	Yes, diffuse
75/I	NOS	Enlarged glomeruli	13	2	2	10	No	0	0–1	Yes, focal
142/I	NOS	N	7	2	1	< 10	No	X	1	Yes, focal

DM GP, diabetic glomerulopathy; MF, marginal fragment; PH, perihilar; TL, tip lesion; NOS, not otherwise specified as per the Columbia classification; info., inflammation; cv, arterial fibro intimal thickening (used primarily in transplant pathology as transplant arteriopathy); AH, arteriole hyalinosis.

The morphology of FSGS in kidney biopsy samples of the patients with two pathogenic variants in the *NPHS2* gene was more pronounced, with a higher portion of globally sclerosed glomeruli in comparison to the patients with a heterozygous variant. Moreover, in the kidney biopsy samples of two patients, both compound heterozygous for *NPHS2* mutations, two types of sclerotisations were found (tip lesion + perihilar; NOS + perihilar). On the other hand, the combination of two types of sclerotisations was not detected in any patient with just one *NPHS2* mutation. The electron microscopic evaluation of kidney biopsies established diffuse foot process effacement in all patients with “genetic FSGS,” while two out of five (40%) patients with one *NPHS2* mutation showed just the focal foot process effacement. Vascular changes in the kidney biopsy samples of all patients were mild.

## Discussion

Genetic FSGS is caused by pathogenic variants in one of the approximately 50 genes associated with a group of proteinuric renal diseases (13, 21). Previous studies, analyzing mostly pediatric FSGS patients manifesting as steroid-resistant nephrotic syndrome (SRNS), identified the *NPHS2* gene as the most frequently mutated gene (22–24) in its genetic forms. The present study, utilizing comprehensive ES and WGS together with advanced bioinformatic analyses, aimed to identify the spectrum of the *NPHS2* pathogenic variants and the clinical phenotype of adult FSGS patients carrying *NPHS2* mutations within a representative Czech cohort of adult cases with histologically verified FSGS.

We identified 3% of patients carrying homozygous or compound heterozygous pathogenic variants in the *NPHS2* gene. Interestingly, 86% of these patients carried the c.868G > A *NPHS2* variant in the homozygous or compound heterozygous state. Moreover, we identified another 2.6% of patients as heterozygous carriers of the c.868G > A *NPHS2* variant. The allelic frequency of the c.868G > A variant within the FSGS cohort was 2.67%. The allelic frequency of the c.868G > A variant among the unrelated patients carrying two *NPHS2* mutations in our FSGS cohort was highly significant in 6 out of 12 alleles (50% allele frequency). The occurrence of this *NPHS2* variant was formerly ascribed to Central and Eastern European FSGS pediatric patients while being absent in Italian and French patients (25). According to the latest data drawn from the Genome Aggregation Database (gnomAD; https://gnomad.broadinstitute.org/news/2018-10-gnomad-v2-1/), the frequency of the c.868G > A mutation in the European non-Finnish population was 0.01093%.

Comparing the frequency of this mutation in Czechia with the one in other geographical regions of Europe, we established that it surpasses the previously published allele frequencies in cohorts of SRNS adolescent and adult patients from other European countries, including Central Europe. For example, the PodoNet cohort gives an allele frequency of 1.1% (one Turkish patient with heterozygous mutation and one German patient with compound heterozygous mutation), while being absent in the French cohort (11, 23, 25–27). In the pediatric SRNS cohort in Poland, just 1 of 40 patients with *NPHS2*-associated SRNS carried the c.868G > A *NPHS2* variant in the heterozygous state (28). A large-scale project analyzing allele frequencies of *NPHS2*-associated SRNS pediatric patients identified the allele frequency of c.868G > A *NPHS2* at 2.6% alleles (13/494 alleles) (15).

Finding 50% allele frequency of the c.868G > A *NPHS2* variant within the patients with *NPHS2*-associated FSGS in our cohort suggested the founder effect that has enriched the frequency of this mutation in the Czech population. We confirmed the founder effect of this mutation by computational enrichment methods and haplotype analysis showing identical haplotypes surrounding the c.868G > A mutation within at least an approximately 0.7 Mb region. Such overrepresentation of the c.868G > A variant in adult FSGS patients could, at least partially, be explained by the late onset of the FSGS disease, often occurring after 18 years of age, and the mild course of disease progression without edemas, thus being likely underdiagnosed in childhood. Such patients can therefore become initially diagnosed only in adulthood and are missing in the pediatric SRNS cohorts.

The proteinuric disease caused by autosomal recessive *NPHS2* mutations usually starts in childhood with early onset SRNS and FSGS. Pediatric patients commonly present with SRNS before the age of 6 years (23) and progress rapidly into ESRD, within the first decade of their life, with low recurrence frequency after kidney transplantation (29). *NPHS2* mutations are also a common cause of congenital and infantile SRNS (13, 23). The p.Val290Met variant expands the clinical phenotypic spectrum of the variants causing *NPHS2* mutations-associated SRNS/FSGS with a much milder phenotype. In our study, patients carrying the p.Val290Met variant in homozygous or compound heterozygous constitution exhibited a relatively late onset of proteinuria, mostly without edema, and had a mild course of the disease. Half of the subjects manifested with proteinuria in childhood and half of the patients in adulthood at a mean age of 27.7 years. Subsequently, two patients (33%) progressed to ESRD at the age of 30 and 37 years, respectively, without post-transplant recurrence. Additionally, four out of six patients (66%), with a mean age of 44 ± 6.27 years, did not progress to renal failure. This phenotype is rather comparable (possibly even milder) to the much less severe clinical phenotype described in patients with SRNS/FSGS caused by the in *trans* association of a pathogenic *NPHS2* variant and the non-neutral polymorphism c.686G > A (p.Arg229Gln) that causes late-onset SRNS at a mean age of 13 years (range 0–39 years) and progresses to ESRD by the age of 26 years (ranging from 10 to 50 years) (10, 12, 16).

The intrafamilial heterogeneity in the two brothers (patients 209/I and 209/II) carrying the homozygous *NPHS2* c.868G > A mutations was intriguing. Moreover, their father (patient 209/III) and mother, both bearing the *NPHS2* c.868G > A mutation in the heterozygous state, showed a different renal phenotype. The father, with negative ES data for Class 4 and 5 variants in 165 proteinuria-associated kidney disease genes, manifested significant proteinuria at the age of 68 years. The mother had all renal parameters normal and did not consent to further genetic testing. This issue merits further study, as yet unknown co-inherited variants may act as “phenotype modifiers” as reported, for example, in the case of the Alport syndrome associated with a variable degree of proteinuria in different family members and with a variable age of onset of kidney failure (30). We are optimistic that, due to high-quality sequencing data together with the utilization of independent bioinformatic pipelines (i.e., as recommended within our participation in the Solve-RD.eu consortium) applied in the analysis of 165 proteinuria-associated kidney disease genes, the VUS are accurately detected and annotated as per the current state-of-the-art technology in the field of medical genomics (gene-specific data are available upon request). Therefore, we anticipate that such variants, thus far being labeled as VUS, may serve as a background for further studies of this kind to facilitate the eventual identification of such modifiers and/or oligogenic inheritance patterns together with long-range sequencing strategies, which will be able to identify haplotypes and eventually utilize telomere-to-telomere reference samples.

According to the study by Bouchireb et al. (17) the compound heterozygosity of the *NPHS2* mutation and polymorphism p.Arg229Gln is the most frequent genotype in adult FSGS patients in Western Europe. However, we did not observe an enrichment of this genotype in our adult FSGS cohort, as the most prominent genotype was homozygosity or compound heterozygosity of the p.V290M variant. The p.Val290Met mutations seem to be associated with the different genetic architecture in our Central European population. We identified only one subject with the p.Arg229Gln polymorphism combined with the p.Ala301Asp *NPHS2* mutation causing FSGS despite the very high frequency of p.Arg229Gln polymorphism that we detected in 13% of patients in our FSGS cohort. This is in line with the study by Tory et al., identifying a significantly lower prevalence of SRNS secondary to the p.Arg229Gln/NPHS2 mutation genotype even when the allele frequency of p.Arg229Gln polymorphism is 15x higher (2.7%) compared to the allele frequency of pathogenic *NPHS2* variants (0.18%) (15).

In conclusion, this study enabled us to identify the most prevalent pathogenic c.868G > A (p.Val290Met) *NPHS2* variant in Czech adult FSGS patients as a founder mutation stemming from the Central European geographic region. We broadened the clinical and histopathological phenotype spectrum of *NPHS2*-associated FSGS in adult patients, which was relatively benign in comparison to early-onset NPHS2-associated SRNS and more similar to p.Arg229Gln variant-associated SRNS. Nevertheless, even the c.868G > A-associated FSGS patients are at risk of underdiagnosis of their disease in childhood and have a higher risk of ESRD in their midlife, and therefore should be longitudinally followed up by a nephrologist.

## Data availability statement

The original contributions presented in the study are publicly available. This data can be found here: https://www.ebi.ac.uk/ena/browser/view/PRJEB67550.

## Ethics statement

The studies involving humans were approved by the Internal Ethics Board of the Motol University Hospital and the 2nd Faculty of Medicine, Charles University in Prague. The studies were conducted in accordance with the local legislation and institutional requirements. The participants provided their written informed consent to participate in this study.

## Author contributions

DT: Conceptualization, Formal analysis, Investigation, Writing—original draft, Writing—review & editing, Methodology, Project administration, Supervision, Validation. MZ: Conceptualization, Formal analysis, Investigation, Writing—review & editing. ML: Conceptualization, Formal analysis, Investigation, Methodology, Validation, Writing—review & editing. JG: Conceptualization, Formal analysis, Investigation, Methodology, Software, Writing—review & editing. PV: Conceptualization, Formal analysis, Investigation, Methodology, Writing—review & editing. SR: Conceptualization, Formal analysis, Investigation, Writing—review & editing. KK: Conceptualization, Formal analysis, Investigation, Writing—review & editing. JR: Conceptualization, Formal analysis, Investigation, Writing—review & editing. EJ: Conceptualization, Formal analysis, Investigation, Writing—review & editing. JM: Conceptualization, Formal analysis, Investigation, Writing—review & editing. MKolláro: Conceptualization, Formal analysis, Investigation, Writing—review & editing. IR: Conceptualization, Formal analysis, Investigation, Writing—review & editing. MHa: Investigation, Writing—review & editing, Conceptualization, Formal analysis. MHo: Conceptualization, Formal analysis, Investigation, Writing—review & editing. MP: Formal analysis, Investigation, Methodology, Writing—review & editing. RŠ: Conceptualization, Formal analysis, Investigation, Writing—review & editing. MKollár: Conceptualization, Formal analysis, Investigation, Writing—review & editing. MM: Conceptualization, Formal analysis, Methodology, Supervision, Writing—review & editing.
